# Pathologic Action of Arsenicals on the Adrenals

**Published:** 1916-06

**Authors:** 


**Pathologic Action of Arsenicals on the Adrenals.** Brown & Pearce, Jour. of Experimental Med., Nov. 1915 concluded from studies on guinea pigs that congestion, haemorrhage, disturbance of lipoid contents, cellular degenerations and necroses and reduction in chromaffin occur, varying in degree according to the chemic constitution of the arsenical, as well as dose. Note: The therapeutic use of any foreign, toxic substance, even if generally advocated and producing immediately favorable results, should be regarded with conservatism. The ultimate results in various structures may be very serious but, on account of the insidiousness of the effect, difficult of recognition. The maximum life of man, aside from rare exceptions, is only four times the length of the growing period. Many lower animals have a life of nearly 10 times this period. It is not impossible that the high mortality of middle aged and moderately old persons is due in large measure to early therapeutic tinkering.

